# *In vitro* antimicrobial potential and phytochemical analysis of root extracts of *Rumex abyssinicus* from southern Ethiopia

**DOI:** 10.1371/journal.pone.0358267

**Published:** 2026-09-11

**Authors:** Gemechu Ameya, Aseer Manilal, Mujib Abdulkadir, Tegenu Gelana, Kuzhunellil Raghavanpillai Sabu

**Affiliations:** 1 Department of Microbial Sciences and Genetics, College of Natural and Computational Sciences, Addis Ababa University, Addis Ababa, Ethiopia; 2 Department of Medical Laboratory Science, Komar University of Science and Technology, Sulaymaniyah, Kurdistan region, Iraq; 3 Department of Chemistry, College of Life Sciences, Arba Minch University, Arba Minch, Ethiopia; China Academy of Chinese Medical Sciences Institute of Chinese Materia Medica, CHINA

## Abstract

The escalating challenge of antimicrobial resistance require a continuous search for novel compounds with therapeutic potential. The approach of linking traditional practices and knowledge with evidence-based preclinical studies can help minimize the resource-intensive process of drug screening. *Rumex abyssinicus* is an important plant species used in Ethiopian traditional medicine, and this study aims to conduct a phytochemical analysis of root extracts of *R. abyssinicus* and determine its antimicrobial activity. *R. abyssinicus* roots were extracted in six solvents of different polarities. Agar well-diffusion assays of the extracts were performed against type culture bacterial strains, clinically bacterial isolates, and fungi, while the tube dilution method was used to determine the minimum inhibitory concentration of the extracts. The ground root powder was refluxed with acetonitrile (1:10 w/v) and analyzed using reverse-phase high-performance liquid chromatography combined with mass-spectrometry. A one-way analysis of variance followed by post hoc multiple comparisons was performed, and differences were considered statistically significant at p < 0.05. The root extract of *R. abyssinicus* showed antimicrobial activity against type culture bacteria and clinical isolates, and fungi. Extraction with different solvents yielded extracts with varying antimicrobial activities. The extracts showed a varied range of antimicrobial activities against the test organisms, with inhibition zones of 11–25 mm for bacteria and 9–24 mm for fungi. The MIC range fell between 12.5 and 100 mg/mL. The phytochemical analysis revealed the presence of major compounds such as emodin (30%), chrysophanol (18%), physcion (16%), helminthosporin (12%), citreorosein (11%) and emodic acid (8%) which are envisaged to have functional roles in chemical defense against the tested organisms. The susceptibilities of the tested bacteria and fungi varied depending on the type of solvent used for extraction and extract concentration. Enhanced antimicrobial activities were observed against the entire panel of bacteria compared to those against the test fungi. The results support the traditional use of *R. abyssinicus* for treating infectious diseases.

## Introduction

The irrational use of antimicrobial agents and the resulting spread of antimicrobial resistance present considerable challenges to the modern healthcare system [1]. Antimicrobial resistance is now recognized as a silent pandemic of the 21st century [2]. An urgent need to develop novel antimicrobial agents exists against the backdrop of rising antimicrobial resistance worldwide. Increasing resistance among bacterial and fungal pathogens is diminishing the efficacy of currently available regimens and poses a substantial threat to global public health [3]. More than ten thousand patients die each day as a result of infections caused by drug-resistant bacteria [4]. Beyond its health implications, AMR imposes a heavy economic burden, resulting in losses of billions of dollars annually [5].

Besides, conventional antimicrobial drugs are often associated with adverse effects, and their limited accessibility in low-income settings has driven research towards safer and more affordable alternatives derived from medicinal plants [5,6]. Traditional medicines serve as an alternative option and continue to play an effective role in disease prevention and management in many regions. The sources of traditional medicines, generally plants, remain integral to healthcare and nutrition globally, especially in developing countries where such formulations constitute a primary component of healthcare systems [7]. Historically, plant-derived medicines have been extensively utilized to manage diverse diseases, and a considerable proportion of modern therapeutics are derived from natural product leads [8,9].

The genus *Rumex* (Polygonaceae) has been extensively used in traditional medicine for the treatment of constipation, inflammation, diarrhoea, wounds, jaundice, skin disorders, tumours, and infections [10]. The genus comprises approximately 200–250 species of annual, biennial, and perennial herbs that are distributed worldwide, particularly in the Northern Hemisphere [11].

*Rumex abyssinicus* is a perennial herb native to the Arabian Peninsula and East Africa, where it is commonly known as “Dhangaggoo” (in Afan Oromo) or Embuacho (in Amharic) in Ethiopia. It has long been used in traditional medicine for the treatment of wounds, diarrhea, dysentery, stomach ache, pharyngitis, typhus, rabies, and inflammatory disorders in both humans and livestock [11]. In Ethiopia, the root of *R. abyssinicus* is widely applied topically for wound healing by traditional healers across several regions. Despite its widespread use in ethnomedicine for treating infections and inflammatory conditions, further studies are required to identify its chemical constituents and elucidate their relationship to its medicinal value [12].

Some of the previous studies have demonstrated that *R. abyssinicus* exhibits significant antioxidant, antimicrobial, anti-inflammatory, and antiviral activities, including activity against influenza A virus [13]. Phytochemical analyses indicate that flavonoids and coumarins are among the major bioactive constituents of the plant [14]. Moreover, *R. abyssinicus* has been reported as a potent inhibitor of urease, an enzyme implicated in several pathological conditions [15]. So far, no study has been conducted in the study area using reverse-phase high-performance liquid chromatography to analyze root extract of the *R. abyssinicus* and evaluate their antimicrobial activity against a wide range of pathogenic microorganism*.* In this study, we specifically focused on investigating the antimicrobial properties and conducting a phytochemical analysis using reverse-phase high-performance liquid chromatography of root extracts of *R. abyssinicus*, which is commonly used by the local community.

## Materials and methods

### Root sample collection

The root samples of *R. abyssinicus* were collected from Chencha, Arba Minch. The plant material was well characterized in the Flora of Ethiopia [16], and the plant material was identified by botanist Garuma Gerbaba Chemeda (PhD) and deposited in the National Herbarium of Addis Ababa University (GG. 28). Chencha is located in the Gamo Gofa Zone, 37 km north of Arba Minch, Ethiopia. Chencha is located at 6°15′N 37°34′E with an elevation of 2,732 m above sea level, providing a cool and relatively moist environment compared with the surrounding lowland areas. The area is predominantly a rural highland district, inhabited mainly by a community that largely depend on subsistence agriculture and long-established traditional practices involving medicinal plant. Average temperatures typically range from 15°C to 22°C, and annual precipitation is estimated to be between 1,000 and 1,600 mm. These environmental conditions support dense vegetation and create favorable habitats for a wide diversity of plant species, including a rich diversity of medicinal plants which are widely used by local communities as part of their traditional healthcare system [17,18].

### Study design

This study employed an integrated *in vitro* pre-clinical experimental design. The crude root extracts of *R. abyssinicus* were evaluated for their antibacterial and antifungal activities using agar well-diffusion assays and determination of minimum inhibitory concentrations (MICs).

### Collection and extraction of *R. abyssinicus* roots

The *R. abyssinicus* plant material was taxonomically authenticated by a qualified plant taxonomist. The collected roots were chopped into small pieces, thoroughly washed under running tap water, rinsed twice with sterile distilled water, and oven-dried at 40°C. The dried material was ground into a fine powder. An amount of 100 g of powder was then soaked separately in 1,000 mL of each solvent for 30 minutes and placed on an orbital shaker at 100 rpm for 24 hours at room temperature. The extracts were then filtered using a sterilized Whatman No. 1 filter paper, and the solvents were removed using a rotary vacuum evaporator at 40°C (Yamato RE 801, Japan) with a round-bottom flask fitted with water condenser. The filtrate obtained were concentrated under reduced pressure in a rotary evaporator at 40°C. After appropriate adjustments and standardizations, the ratios of the respective solvents to the weight of the crude extracts were determined. The color of the obtained extract varied depending on the solvent used for extraction. The resulting residues were weighed and reconstituted in respective solvents to obtain a stock concentration of 100 mg/mL and stored at −20 °C until further use [19,20]. For reverse-phase high-performance liquid chromatography-mass spectrometry (RPHPLC-MS) analysis, the above-mentioned procedure was performed to obtain the extract using acetonitrile as the solvent.

### Culture and maintenance of test microorganisms

The antimicrobial activities of root extracts were evaluated against multiple panels of microorganisms. The first panel consisted of pure cultures of ATCC standard organisms (*Staphylococcus aureus*, *Salmonella enterica*, *Klebsiella pneumoniae,* and *Escherichia coli*), procured from the Ethiopian Public Health Institute. The second panel consisted of clinically isolated bacteria, including WHO-priority bacterial pathogens: methicillin-resistant *Staphylococcus aureus* (MRSA), extended-spectrum β-lactamase (ESβL) producing *E. coli,* piperacillin-resistant *Pseudomonas aeruginosa* (PRP) [17], and diarrheagenic clinical isolates of *Shigella dysenteriae*. The third panel consisted of clinically isolated fungi (*Candida albicans, Penicillium* sp.*, Aspergillus flavus,* and *A. niger*). All clinical isolates were obtained from Arba Minch General Hospital and were transported in triple-layer packaging under aseptic conditions. Bacterial isolates were maintained on nutrient agar, while fungal isolates were maintained on Sabouraud dextrose agar (SDA). Subculturing was performed regularly, and cultures were stored at 4°C until use. All experiments were conducted under appropriate biosafety conditions, with appropriate disinfection procedures.

### Agar well-diffusion assay

Antibacterial and antifungal activities were evaluated using Mueller–Hinton agar (MHA) and SDA, respectively, following the Clinical and Laboratory Standards Institute guidelines [21]. A standardized 0.5 McFarland suspension of each test organism was evenly spread on the agar surface. Diffusion wells of approximately 6 mm diameter were prepared using a sterile 200 µL pipette tip (yellow), and 20 µL of each extract at a concentration of 50 mg/mL was added separately to each well. The respective solvents served as negative controls. Inoculated Petri plates were incubated at 37°C for 24 h for bacteria and 48 h for fungi [22].

### Determination of minimum inhibitory concentrations

The antibacterial activities of root extracts were evaluated by determining the minimum inhibitory concentrations using the tube dilution technique with nutrient broth. Two-fold serial dilutions of each extract, prepared in the respective solvents, were made to produce concentrations ranging from 1.56 to 100 mg/mL. The initial tube contained a mixture of double-strength nutrient broth and the root extract suspension, from which subsequent serial dilutions were prepared. Each diluted extract was aseptically inoculated with bacterial cultures adjusted to the turbidity of a 0.5 McFarland standard and incubated at 37 °C for 24 hours. Following incubation, the lowest concentration that showed no visible bacterial growth was recorded as the MIC. Control tubes containing nutrient broth inoculated with the test organisms, with and without the solvent used for extraction, were also included [17,23]. The MIC determination procedures were performed in accordance with the CLSI guidelines [21].

In the case of fungal isolates, MICs were determined by the agar dilution method. Stock solutions of the crude root extracts were mixed with sterile molten Mueller–Hinton agar and Sabouraud dextrose agar, cooled to approximately 45 °C. Two-fold serial dilutions were then prepared to achieve final concentrations ranging between 1.56 and 100 mg/mL, as in the case of bacterial isolates. The resulting agar–extract mixtures were poured into sterile Petri dishes and allowed to solidify. Each plate was inoculated with a fungal suspension standardized to a 0.5 McFarland turbidity and incubated at 37 °C for 48 hours. The MICs were identified as the lowest concentrations of the extracts that completely inhibited the visible growth of the tested fungi; SDA plates containing the fungus with and without the extraction solvent, served as controls [17,23].

### Reverse-phase high-performance liquid chromatography

The ground root powder was refluxed with acetonitrile (1: 10 w/v) was analyzed using reverse-phase high-performance liquid chromatography (RP-HPLC) combined with mass-spectrometry (MS) (Waters Alliance 2695). The negative ionization mode (ESI 10–40 eV) was selected to identify the fragments produced using a nonpolar Waters symmetry C18 column (250 X 4.6 mm; 5.0µm). The mobile phase consisted of an acetonitrile-water mixture (80:20 v/v) buffered to pH 4.5 with acetate buffer and trifluoroacetic acid, applied isocratically at ambient temperature. The analysis was conducted with a flow rate of 1 ml/minute and a pressure of 4000 psi, utilizing an injection volume of 45 µL for the analyte, which was prepared at a concentration of 1 µg/mL in an 80:20 v/v acetonitrile-water mixture [24].

### Quality control

All reagents and culture media were checked for their expiration dates before use. Extracts and inoculated media were stored at 2–8 °C; laboratory procedures strictly adhered to the in-house standard operating procedures for pre-analytical, analytical, and post-analytical quality assurance procedures. The agar well diffusion assay were done in triplicate to minimize measurement bias.

### Data processing and statistical analysis

All agar diffusion assay experiments were conducted in triplicate; data were presented as the mean ± standard deviation (SD), and statistical analysis was performed using SPSS version 25. One-way analysis of variance (ANOVA), followed by post-hoc multiple comparison tests, was used to evaluate differences among groups. Statistical significance was set at P < 0.05.

### Ethics statement

Ethical approval for this study was obtained from the College of Natural and Computational Sciences, Addis Ababa University, by Gemechu Ameya. This study does not involve human subjects or human tissues; rather, it involves only isolated pathogenic microorganisms. For the clinically isolated bacterial and fungal test organisms, the sources were de-identified and were not disclosed; only the isolated microorganisms were used in the study. All procedures were conducted in accordance with institutional ethical standards, standard laboratory protocols, and relevant microbiological research guidelines.

## Results

### Characteristics of root extracts

The crude root extracts obtained using acetone, chloroform, ethyl acetate, ethanol, methanol, and water exhibited noticeable differences in extraction yield and physical appearance. The extracts varied in color from light yellowish-brown to dark brown, with differences in consistency ranging from low viscous to viscous or resinous masses. The methanol, ethanol, and aqueous extracts generally appeared darker and more viscous, whereas the chloroform and ethyl acetate extracts were lighter in color with relatively less viscous consistency. The acetone extract exhibited an intermediate appearance. Differences were also observed in texture and odor among the extracts.

### Agar well diffusion assays against bacteria

**Fig 1:**
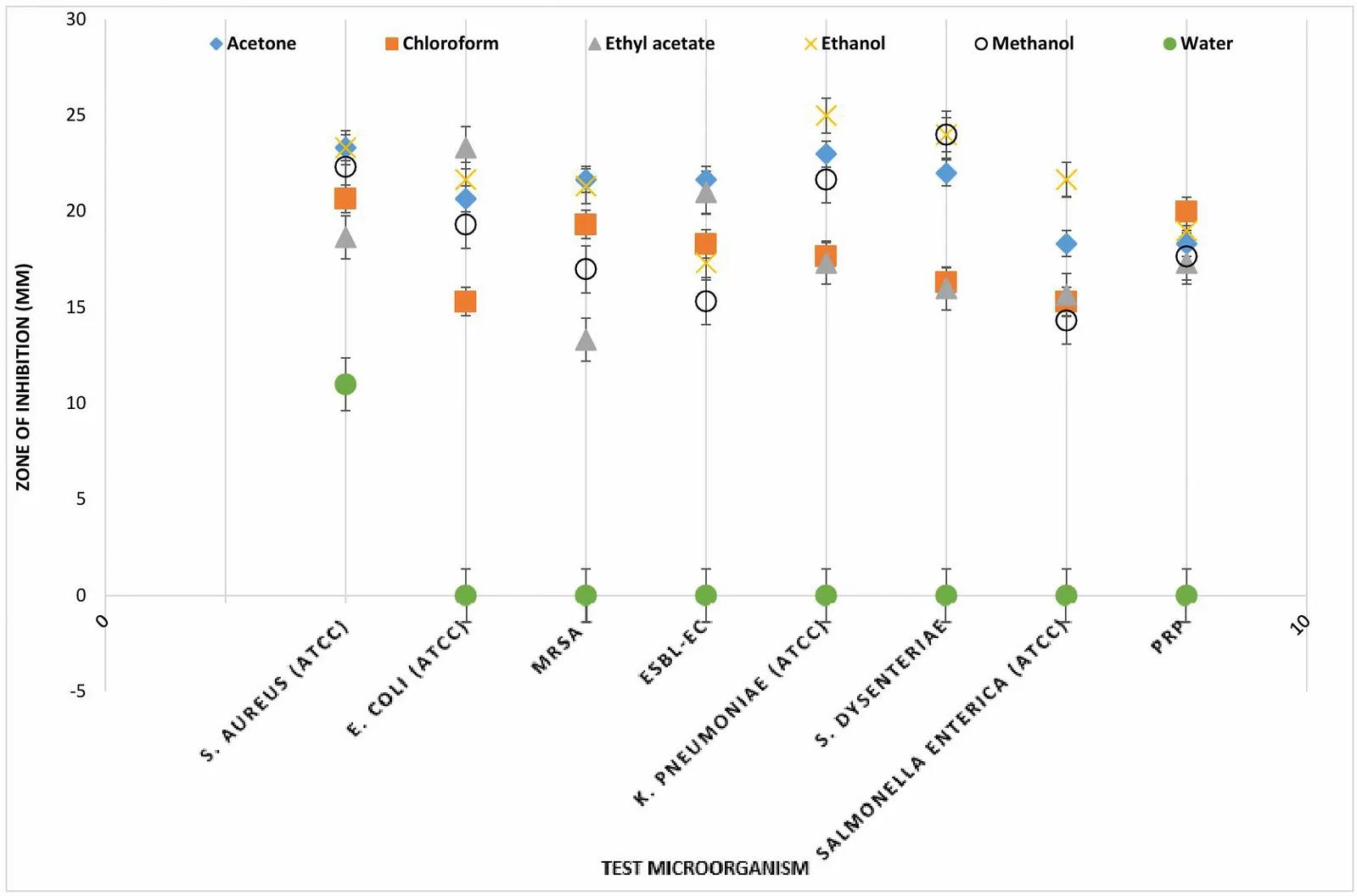
Overall antimicrobial activity of different solvent extracts against selected test organisms based on agar diffusion assay.

The antimicrobial activity of *R. abyssinicus* root extract varies considerably depending on the solvents used, with the ethanol extract demonstrating the relatively highest and broadest activity across the tested bacterial strains. The acetone extract exhibited relatively consistent antimicrobial activity across the different test organisms compared with the other solvent extracts. In contrast, the antimicrobial activities of the ethyl acetate and ethanol extracts varied considerably depending on the test organism. The aqueous extract demonstrated the lowest antimicrobial activity against most of the test organisms at the same extract concentration (Fig 1).

The ethanolic extracts showed considerable antimicrobial activity against the drug-resistant clinical isolates, including MRSA (21 mm), EsβL-EC (17 mm) and PRP (19 mm). This extract produced the largest zone of inhibition (25 mm) against the type culture of *K. pneumoniae*, which was identified as the most susceptible bacterium, while consistently generating larger inhibition zones against other species. The acetone extract also demonstrated strong antimicrobial activity, particularly against the type culture of *S. aureus* (23 mm) and *K. pneumoniae* (23 mm), with no significant difference from the ethanol extract. Moderate activities were observed for the chloroform and ethyl acetate extracts, which produced intermediate zones of inhibition. In contrast, the aqueous extract exhibited significantly lowest activity (P < 0.001), failing to inhibit the growth of most of the tested bacteria species and showing only minimal activity against *S. aureus* (11 mm). Among the organisms tested, *Salmonella enterica* appeared to be the most resistant to the organic extracts, consistently recording the smallest inhibition zones, including the lowest observed value of 14 mm for the methanol extract. This was followed by ESβL-EC and PRP, which also showed relatively lower susceptibility to the extracts. Overall, the acetone and ethanol extracts of the *R. abyssinicus* exhibited the greatest antimicrobial effectiveness, followed by the methanol, chloroform, and ethyl acetate extracts, whereas the aqueous extract showed poor activity (Table 1).

**Table 1:** Antimicrobial activity of *R. abyssinicus* root extract against type culture and clinical isolate bacteria by agar well diffusion assay.

Bacteria species	Zone of inhibition (mm)					
	Acetone ^a^	Chloroform^b^	Ethyl acetate^b^	Ethanol ^a^	Methanol ^ab^	Water ^c^
*S. aureus* (ATCC)	23.33 ± 1.53	20.67 ± 0.58	18.67 ± 2.08	23.33 ± 0.58	22.33 ± 0.58	11.00 ± 1.00
*E. coli* (ATCC)	20.67 ± 2.08	15.33 ± 0.58	23.33 ± 1.53	21.67 ± 2.08	19.33 ± 2.08	–
*MRSA*	21.67 ± 1.53	19.33 ± 0.58	13.33 ± 1.53	21.33 ± 1.53	17.00 ± 0	–
EsβL-EC	21.67 ± 1.53	18.33 ± 1.53	21.00 ± 1.00	17.33 ± 1.53	15.33 ± 1.53	–
*K. pneumoniae* (ATCC)	23.00 ± 0	17.67 ± 1.53	17.33 ± 2.08	25.00 ± 1.00	21.67 ± 1.53	–
*S. dysenteriae*	22.00 ± 2.00	16.33 ± 1.15	16.00 ± 1.00	24.00 ± 2.00	24.00 ± 1.00	–
*Salmonella enterica* (ATCC)	18.33 ± 0.58	15.33 ± 1.15	15.67 ± 1.15	21.67 ± 1.53	14.33 ± 0.58	–
PRP	18.33 ± 0.58	20.00 ± 1.00	17.33 ± 0.58	19.00 ± 1.00	17.67 ± 0.58	–

MRSA: Methicillin-resistant *Staphylococcus aureus*; ESβL-EC: extended spectrum β-lactamase producing *E. coli*; PRP: piperacillin-resistant *Pseudomonas aeruginosa*. The ^**a, ab, b**^ indicate relative difference among the non-aqueous solvent extract, while ^**c**^ indicates significant difference in antimicrobial activity between the aqueous and non-aqueous extraction solvents (P < 0.05).

### Antifungal activity of the extract using agar well diffusion assay

The antifungal activities of *R. abyssinicus* root extracts were tested against four clinically isolated fungal species (*Candida albicans, Penicillium* sp.*, A. niger,* and *A. fumigatus*) (Table 2). The efficacy of the extract varied depending on the type of solvent used for extraction and the tested fungal species. Overall, there was a significant difference between the aqueous and non-aqueous extracts (P < 0.001). Among the non-aqueous extracts, the acetone-based extract produced the highest inhibition zone (24 mm) against *C. albican*s followed by the ethanol extracts. Chloroform and methanol extracts showed moderate activity, with zones of inhibition ranging from 14.67 to 22 mm. The lowest activity among the non-aqueous extracts was observed with the ethyl acetate extract, with the smallest inhibition zones, ranging from 9.67 to19.33 mm. On the other hand, the aqueous extract showed significantly lower antifungal activity, with activity observed only against *C. albican*s. Acetone produced the highest level of inhibition, while chloroform and ethanol demonstrated similar effects. Overall, organic solvents produce greater antifungal activity than water (Table 2).

**Table 2:** Antimicrobial activity of *R. abyssinicus* root extract against clinical isolate fungal species by agar well diffusion assay.

Fungal species	Zone of inhibition (mm)					
	Acetone ^a^	Chloroform ^ab^	Ethyl acetate ^b^	Ethanol ^ab^	Methanol ^b^	Water ^c^
***C. albicans***	24.00±1.00	22.33±0.58	19.33±1.15	22.33±0.58	20.33±1.53	9.00±1.00
***Penicillium* sp.**	17.33±1.53	15.67±2.08	11.67±1.53	17.33±1.53	14.67±1.53	-
***A. niger***	14.67±1.53	17.00±1.73	9.67±1.53	20.67±2.08	14.00±2.00	-
***A. fumigatus***	15.00±2.00	15.33±1.53	14.00±2.00	18.33±1.53	15.67±1.53	-

The ^**a, ab, b,**^ indicate relative difference among the non-aqueous solvent extract, while ^**c**^ indicates significant difference in antimicrobial activity between aqueous and non-aqueous extraction solvents (P < 0.05).

### Minimum inhibitory concentrations of the root extracts

The minimum inhibitory concentrations (MICs) of all non-aqueous extracts were determined. Among bacteria isolates, the lowest MIC recorded was 12.5 mg/ml. Acetone and ethanol extracts exhibited the strongest antimicrobial activity in the MIC assay. Among the tested bacteria, *S. aureus*, *K. pneumoniae*, and *S. dysenteriae* were the most susceptible to the extracts, whereas *S. enterica* was the least susceptible to both extracts. Consistent with the agar diffusion assay, the chloroform and ethyl acetate extracts exhibited relatively low antimicrobial activities in the MIC assay. Overall, *Salmonella enterica,* and *E. coli* were the least susceptible bacteria to the non-aqueous extracts (Table 3).

**Table 3:** Minimum inhibitory concentration of *R. abyssinicus* root extracts against test organisms.

Test organisms category	Species of test organism	MIC (mg/mL)				
		Acetone	Chloroform	Ethyl acetate	Ethanol	Methanol
Bacteria	*S. aureus* (ATCC)	12.50	25.00	50.00	12.50	12.50
	*E. coli* (ATCC)	50.00	50.00	25.00	25.00	50.00
	*MRSA*	25.00	25.00	100.00	50.00	50.00
	EsβL-EC	50.00	50.00	25.00	25.00	100.00
	*K. pneumoniae* (ATCC)	12.50	25.00	100.00	12.50	12.50
	*S. dysenteriae*	25.00	100.00	100.00	12.50	12.50
	*Salmonella enterica* (ATCC)	50.00	50.00	100.00	50.00	100.00
	PRP	50.00	25.00	50.00	25.00	50.00
Fungi	*C. albicans*	12.50	12.50	25.00	12.50	25.00
	*Penicillium* sp.	25.00	100.00	*	100.00	50.00
	*A. niger*	100.00	50.00	*	50.00	100.00
	*A. fumigatus*	50.00	100.00	*	50.00	50.00

MRSA: Methicillin-resistant *Staphylococcus aureus*; ESβL-EC: extended spectrum β-lactamase producing *E. coli*; PRP: piperacillin-resistant *Pseudomonas aeruginosa*. MIC: Minimum inhibitory concentration. * MIC > 100 mg/mL

Regarding the clinical fungal isolates, the lowest MIC (12.5 mg/ml) was observed against *C. albicans* whereas *A. niger* and *A. fumigatus* were resistant to the extract. Overall, the acetone extract demonstrated the greatest antifungal activity, with MIC values of 12.5 mg/mL against *C. albicans* and 25 mg/mL against *Penicillium* sp. Ethanol and chloroform extracts also exhibited good activity against *C. albicans*, with each showing an MIC of 12.5 mg/mL. However, both extracts exhibited substantially weaker activity against *Penicillium* sp., with an MIC value of 100 mg/mL. The ethyl acetate extract was the least effective against the fungal isolates, with MIC values exceeding 100 mg/mL for three of the four fungal species tested (Table 3).

### Phytochemical analysis of the *R. abyssinicus*

The relative percentage of the compounds identified in the root extract of the plant are summarized in Table 4. Analysis of the acetonitrile extract of *R. abyssinica* revealed that the major phytoconstituents were emodin (30%), chrysophanol (18%), physcion (16%), helminthosporin (12%), citreorosein (11%), and emodic acid (8%).

**Table 4:** Phytochemical analysis of active fractions of *R. abyssinicus* root extract.

SI no	Compound name	MF	MW	Mass fragmentation (m/z)	W/W (%)	Collision Energy (eV)	Functionality	Formula Name
1	Emodin	C_15_H_8_O_5_	270	269; [M-H]^–^ 225 [M-H-CO_2_]^–^	30	20	Trihydroxy methyl anthraquinone	1,3,8-trihydroxy-6-methyl anthraquinone
2	Helminthosporin	C_15_H_10_O_5_	270	269; [M-H]^–^ 254 [M-H-CH_3_]^–^	12	20	Trihydroxy methyl anthraquinone	1,5,8- trihydroxy −3-methyl-anthraquinone
3	Citreorosein	C_15_ H_10_O_6_	286	285; [M-H]^–^ 267 [M-H-H_2_O]^–^ 241 [M-H-CO_2_]^–^ 225 [M-H-CO_2_^--^H_2_O]^–^	11	21	Trihydroxy methyl anthraquinone	1,3,8-trihydroxy-6-hydroxymethyl-9,10- anthraquinone
4	Chrysophanol	C_15_H_10_O_4_	254	253; [M-H]^–^ 225 [M-H-CO]^–^ 235 [M-H-H_2_O]^–^ 209 [M-H-H_2_O-CO_2_]^–^	18	21	Dihydroxy methyl anthraquinone	1,8-di hydroxy-3 – methyl – anthraquinone
5	Physcion (parietin)	C_16_H_12_O_5_	284	283; [M-H]^–^ 268 [M-H-CH_3_]^–^ 255 [M-H-CO]^–^ 239 [M-H-CO-CO_2_]^–^	16	25	Methyl ether of Dihydroxy anthraquinone	1,8-Dihydroxy 3- methoxy-6-methyl anthraquinone
6	Emodic acid	C_15_H_8_O_7_	300	299; [M-H]^–^ 255 [M-H-CO_2_]^–^ 227 [M-H-CO_2_ – CO]^–^	8	26	Trihydroxy methyl anthraquinone	1, 6, 8-trihydroxy-3- carboxy- 9, 10- anthraquinone
7	Chrysophanol 8-O-β-d-glucoside	C_21_H_20_O_9_	416	415; [M-H]^–^ 253 [M-H-Glc]^–^ (i.e., loss of glucosyl residue) 225 [M-H-Glc- CO]^–^	2.5	30	Glycosidic derivative of phenolic anthraquinone	8-(β-D-glucopyranosyloxy)-1-hydroxy-3-methyl-9,10-anthracenedione
8	Emodin-8-O-β-d-glucoside	C_21_H_20_O_10_	432	431; [M-H]^–^ 269 [M-H-Glc]^–^	2.5	31	Glycosidic derivative of phenolic anthraquinone	8-(β-D-glucopyranosyloxy)-1-hydroxy-3-methyl-9,10-anthracenedione

## Discussion

Ancient and modern drug discovery approaches face various challenges and limitations. Traditional medicinal plant discovery was largely based on trial-and-error practices involving direct use in humans and animals, with little scientific evaluation of dosage, safety, or toxicity. However, traditional medicine has played a significant role in the development of many modern pharmaceuticals derived from natural products [25]. In contrast, modern drug discovery requires extensive preclinical and clinical investigations, making the process time-consuming and expensive. Another major challenge facing modern medicine is antimicrobial resistance, which occurs when microorganisms develop resistance, reducing the effectiveness of existing drugs [26]. Therefore, approaches that integrate preclinical scientific studies with traditionally used medicinal plants may facilitate drug discovery while minimizing the time and resource investment. In this study, we investigated the antimicrobial activity and phytochemical composition of the well-known traditionally used medicinal plant *Rumex abyssinicus*.

The ethanol extract of *R. abyssinica* showed the highest and broadest activity against the entire panel of bacteria and fungi. It demonstrated maximum inhibition zones against Gram-negative bacteria particularly *K. pneumoniae* and *S. dysenteriae*. This result is more or less similar to previous Ethiopian studies [27,28]. This may be related to the ability of the polar organic solvents in extracting a diverse range of phytochemicals from plant parts in general [13]. In our study, acetone and methanol extracts also showed moderate-to-high activity, specifically against WHO priority bacteria, such as MRSA and ESβL-EC, whereas the aqueous extract exhibited only minimal activity. The primary active constituents, such as the anthraquinones emodin and physcion, are typically extracted more efficiently using polar organic solvents [28].

Among the test microorganisms, *K. pneumoniae* and *S. aureus* were the most susceptible to the extracts, while *S. enterica* and the PRP isolate appeared more resistant. This might be due to the frequent use of the medicinal plant for common infectious diseases such as typhoid fever. Our previous work on *R. nepalensis* showed that the EtOH extract exhibited the greatest inhibition against *E. faecalis*, *E. coli*, and *S. aureus* [17]. This variation may be due to differences in the bacterial strain used in the evaluation of antimicrobial activity; the pattern of drug resistance observed among the tested bacteria and the concentration of the extracts used differed when comparing the two studies. Furthermore, the antimicrobial activity of plants is influenced by several critical factors, such as plant species, season, geographical location, harvesting period, extraction methodology, purity of extracts, and the solvents used.

The medicinal plant extract showed activity against clinically isolated fungal species. Isolates of *C. albicans* were relatively the most susceptible among all the tested fungal species. A similar finding was also observed in a study conducted by Al-Asmari et al. 2015 [29]. The observed susceptibility of *C. albican* is consistent with existing literature, which reports inhibition zones for *R. abyssinicus* against this pathogen ranging from 11 to 29 mm, often attributed to the presence of secondary metabolites such as anthraquinones and chrysophanol [13]. Non-aqueous extracts showed better antifungal activity against the tested yeast and molds while the aqueous extract showed antifungal activity only against the yeast, *C. albican. This may be due to the nature of fungal species, as molds are usually resistant to chemicals and secondary metabolites. Studies have also shown that molds have multiple mechanisms that contribute to resistance against antifungal chemicals and environmental stresses compared with yeasts* [30].

In our study, agar well diffusion assay was supplemented by determination of the minimum inhibition concentration (MIC). MIC values as low as 12.5 mg/ml were observed for *R. abyssinicus* root extract against both bacterial and fungal pathogens in our study. This indicates that the extract has broad-spectrum activity against the test microorganisms which further implies the presence of multiple bioactive phytochemical compounds. The alcoholic extracts were the most effective solvents, particularly against *S. aureus*, *K. pneumoniae*, and *Shigella* sp. (the lowest MIC value being 12.5 mg/ml). This finding is consistent with previous similar studies [26]. In the case of fungal pathogens, the acetone extract showed the lowest MIC, which was 12.5 mg/ml against *C. albicans*. Some other studies have reported MICs between 32 and 64 mg/mL for methanolic extracts against *C. albicans* and *C. neoformans* [28]. This indicates that the medicinal plant has consistent antimicrobial activity accross different geographical location and extraction methods.

RP-HPLC-MS analysis of the acetonitrile root extract of *Rumex abyssinica* identified several bioactive phytochemicals, including emodin, physcion, helminthosporin, chrysophanol, emodic acid, and citreorosein, all of which exhibit considerable antimicrobial activity. Emodin has been reported to possess antibacterial activity against *Mycobacteria*, methicillin-resistant *Staphylococcus aureus* (MRSA), and *Bacillus subtilis*, in addition to antiviral activity against influenza virus [31,32]. Likewise, physcion demonstrates strong antibacterial effects against *Staphylococcus aureus*, *Staphylococcus epidermidis*, and *Pseudomonas aeruginosa*, together with antifungal activity [33]. Helminthosporin, another anthraquinone derivative isolated from *Rumex* species, has also been recognized for its broad-spectrum antimicrobial potential [34]. In addition, chrysophanol contributes significant antibacterial, antifungal, and antiviral activities, including inhibitory effects against poliovirus, coxsackievirus, and rhinovirus [35].

Among these phytochemicals, emodic acid appears particularly promising due to its potent antibacterial activity against Gram-positive bacteria, including drug-resistant strains such as MRSA. Its antimicrobial mechanism is primarily associated with the disruption of bacterial membrane integrity, leading to increased membrane permeability and impairment of essential intracellular processes [36]. Furthermore, emodic acid has been reported to interfere with bacterial biofilm formation, nucleic acid synthesis, protein synthesis, and cellular energy metabolism, ultimately resulting in bacterial cell death [36]. Collectively, the identified anthraquinone derivatives in *R. abyssinica* provide substantial evidence supporting the antimicrobial potential of the plant and highlight its prospective value as a source of novel antimicrobial agents for combating antimicrobial-resistant pathogens.

In our study, we used acetonitrile extraction for phytochemical analysis. Acetonitrile was selected for the reversed-phase chromatographic analysis because it is widely regarded as a preferred organic mobile-phase solvent due to its low UV absorbance, low viscosity, strong elution strength, and ability to produce improved chromatographic peak shapes and resolution across a broad range of phytochemical constituents [37]. Its intermediate polarity also enables the detection of compounds with diverse physicochemical properties that are commonly extracted using solvents of varying polarities. Despite these favorable analytical characteristics, acetonitrile was not used as an extraction solvent for the antimicrobial assays because of potential health risks associated with prolonged exposure. Furthermore, the objective of this study was not to isolate and evaluate the antimicrobial activity of individual phytochemicals or to perform a comprehensive phytochemical comparison of extracts obtained with different solvents. Instead, we selected an intermediate-polarity solvent to provide a representative phytochemical profile while focusing on the primary aim of generating scientific evidence that supports traditional medicinal knowledge with preclinical data. This approach helps bridge traditional knowledge systems with modern scientific validation and may facilitate future drug discovery efforts.

## Conclusion

It can be concluded that the crude root extracts of *R. abyssinicus* possess broad-spectrum antimicrobial properties. Ethanol and acetone extracts were the most effective, demonstrating superior inhibitory activity against the entire panel of tested bacterial and fungal pathogens. The findings highlight their activity against drug-resistant isolates, including MRSA, PRP, and EsβL-EC. The lowest MIC values were recorded for ethanol and acetone extracts against several pathogens. The high antimicrobial potency can be correlated with the synergistic effects of several anthraquinone derivatives, including emodin, chrysophanol, physcion, helminthosporin, citreorosein, and emodic acid. Overall, these results support the traditional medicinal use of *R. abyssinicus* for the treatment of infectious diseases*.* Future studies should focus on investigating the antimicrobial activity of individual isolated compounds, together with comprehensive pharmacokinetic and pharmacodynamic evaluations, to better characterize their therapeutic potential and mechanisms of action.

## Supporting information

S1 Data Agar well diffusion dataset. (XLSX)

S2 Data Minimum inhibitory concentrations dataset. (XLSX)
